# 

**Published:** 1863-05

**Authors:** 


### BOOKS RECEIVED.

*Diseases of Women and Children.* By Gunning S. Bedford. William Wood, New York.

*Wilson on Diseases of the Skin, with plates.* Blanchard & Lea, Philadelphia.

*Cazeaux's Midwifery.* Lindsay & Blakiston, Philadelphia.

*Pain's Institutes of Medicine—seventh edition—revised.* Harper & Brothers, New York.

*Meigs' Treatise on Obstetrics—fourth edition, with one hundred twenty-nine illustrations.* Blanchard & Lea, Philadelphia.

*Packard's Minor Surgery, with one hundred and forty-five illustrations.* J. B. Lippincott & Co., Philadelphia.

*Medical Student's Vade Mecum.* By George Mendenhall, M. D., &c., *with two hundred and twenty-four illustrations.* Philadelphia: Lindsay & Blakiston, 1863.

*Brande & Taylor's Chemistry.* Philadelphia: Blanchard & Lea.

*Bowman's Medical Chemistry.* Philadelphia: Blanchard & Lea.

*The Manual of Electricity for the use of Physicians, Dentists, Students, and the public, being a Treatise of Electricity, and explaining its application to telegraphs, submarine batteries, blasting, embalming, electroplating and typing, galvanizing, decomposing water, extracting metals from the human body, and the cure of many diseases, with ample directions for its use and application—a full statement of diseases in the treatment of which Electricity is now considered useless or injurious. Directions for its use in extracting teeth without pain, and other valuable information.* By H. Lassing, M. D. Published for the author by Samuel B. Smith, 429 Broadway, N. Y.

*Speech of the Rev. Dr. Fellows, President of the United States Sanitary Commission, made at the Academy of Music, Philadelphia, Tuesday evening, February* 24, 1863.

*Sanitary Commission—Supplement to Fourth Report, concerning the Aid and Comfort given by the Sanitary Commission to sick soldiers passing through Washington.* By FREDERICK N. KNAPP, Special Relief Agent.

